# Vorapaxar treatment reduces mesangial expansion in streptozotocin-induced diabetic nephropathy in mice

**DOI:** 10.18632/oncotarget.25069

**Published:** 2018-04-24

**Authors:** Maaike Waasdorp, JanWillem Duitman, Sandrine Florquin, C. Arnold Spek

**Affiliations:** ^1^ Center for Experimental and Molecular Medicine, Academic Medical Center, Amsterdam, The Netherlands; ^2^ Inserm UMR1152, Physiopathologie et Epidémiologie des maladies respiratoires, Medical School Xavier Bichat, Paris, France; ^3^ Département Hospitalo-Universitaire FIRE (Fibrosis, Inflammation and Remodeling) and LabEx Inflamex, Université Paris Diderot, Sorbonne Paris Cité, Paris, France; ^4^ Department of Pathology, Academic Medical Center, Amsterdam, The Netherlands

**Keywords:** diabetic nephropathy, vorapaxar, protease-activated receptor-1, mesangial expansion, streptozotocin, Pathology

## Abstract

**Background:**

Twenty years after the onset of diabetes, up to 40% of patients develop diabetic nephropathy. Protease-activated receptor-1 (PAR-1) has recently been shown to aggravate the development of experimental diabetic nephropathy. PAR-1 deficient mice develop less albuminuria and glomerular lesions and PAR-1 stimulation induces proliferation and fibronectin production in mesangial cells *in vitro*. Vorapaxar is a clinically available PAR-1 inhibitor which is currently used for secondary prevention of ischemic events.

**Objectives:**

The aim of this study was to investigate in a preclinical setting whether vorapaxar treatment may be a novel strategy to reduce diabetes-induced kidney damage.

**Results:**

While control treated diabetic mice developed significant albuminuria, mesangial expansion and glomerular fibronectin deposition, diabetic mice on vorapaxar treatment did not show any signs of kidney damage despite having similar levels of hyperglycemia.

**Conclusions:**

These data show that PAR-1 inhibition by vorapaxar prevents the development of diabetic nephropathy in this preclinical animal model for type I diabetes and pinpoint PAR-1 as a novel therapeutic target to pursue in the setting of diabetic nephropathy.

**Materials and Methods:**

22 C57Bl/6 mice were made diabetic using multiple low-dose streptozotocin injections (50 mg/kg) and 22 littermates served as non-diabetic controls. Four weeks after the induction of diabetes, 11 mice of each group were assigned to control or vorapaxar treatment. Mice were sacrificed after 20 weeks of treatment and kidney damage was evaluated.

## INTRODUCTION

Diabetic nephropathy is a major complication of diabetes mellitus, and the leading cause of end stage renal disease worldwide [1]. Twenty years after onset of diabetes, microalbuminuria –the first sign of diabetic nephropathy- is detected in up to 40% of patients [2–4]. Because of the growing number of diabetes patients in combination with an earlier onset of the disease, the incidence of diabetic nephropathy will likely rise in the coming years. With strict glycemic control and angiotensin-converting-enzyme inhibition it is possible to slow the progression of renal failure, although many patients still eventually progress towards end stage renal disease. At that stage, dialysis or kidney transplantation are the only available treatment options to date. Both dialysis and kidney transplantation have a huge social and economic impact and alternative treatment options are thus eagerly awaited for [5].

Considering the emerging role of coagulation factors in kidney disease (excellently reviewed by Madhusudhan and colleagues [6]), it is tempting to speculate that anticoagulant therapy may be a promising strategy to prevent kidney damage in diabetic patients. Many clinical trials with anticoagulants have indeed been performed [7–12], but enthusiasm for anticoagulants in the setting of diabetic nephropathy faded away due to conflicting results [13, 14]. Interestingly however, the recent notion that coagulation factor receptors, i.e. protease-activated receptors (PARs), are omnipresent in renal cells not only provides a molecular link between coagulation factors and renal cell function but also suggest that targeting PARs may hold therapeutic promise in the setting of diabetic nephropathy.

PAR-1 is a seven transmembrane domain receptor that belongs to the family of G protein-coupled receptors (GPCRs) [15]. In contrast to traditional GPCRs, PAR-1 is activated by proteolytic cleavage instead of ligand binding. Originally, PAR-1 was identified as the thrombin receptor but alternative agonists, such as activated protein C [16], plasmin [17], or metalloprotease 13 [18] have been shown to also activate this receptor - a phenomenon called biased agonism [15, 16]. In the kidney, PAR-1 expression is increased upon diabetes. Activation of PAR-1 in mesangial cells induces their proliferation and leads to the production of extracellular matrix [19]. In line with this potential profibrotic role of PAR-1 during diabetes, we recently showed that PAR-1 deficient mice develop less glomerulopathy during streptozotocin (STZ)-induced type 1 diabetes. Indeed, glomerular cell proliferation and fibronectin deposition was significantly reduced in diabetic PAR-1 deficient mice as compared to diabetic wild type mice. It is consequently tempting to speculate that PAR-1 may be a potential novel target to reduce diabetes-induced kidney damage [19].

Although transgenic mice are perfectly suited to perform mechanistic studies and to identify potential novel targets, translation of preclinical studies using knock out animals remains challenging. Before initiating clinical studies targeting PAR-1 in the setting of diabetic nephropathy it is therefore pivotal to establish whether pharmacological inhibition of PAR-1, started once diabetes has been established, also limits diabetes-induced kidney damage. Consequently, we here evaluated the potential inhibitory effect of vorapaxar (SCH530348, Zontivity), a PAR-1 antagonist used for secondary prevention of atherothrombotic events in patients with previous myocardial infarct, on STZ-induced diabetic nephropathy.

## RESULTS

### Vorapaxar treatment reduces diabetes-induced kidney damage

**Figure 1:**
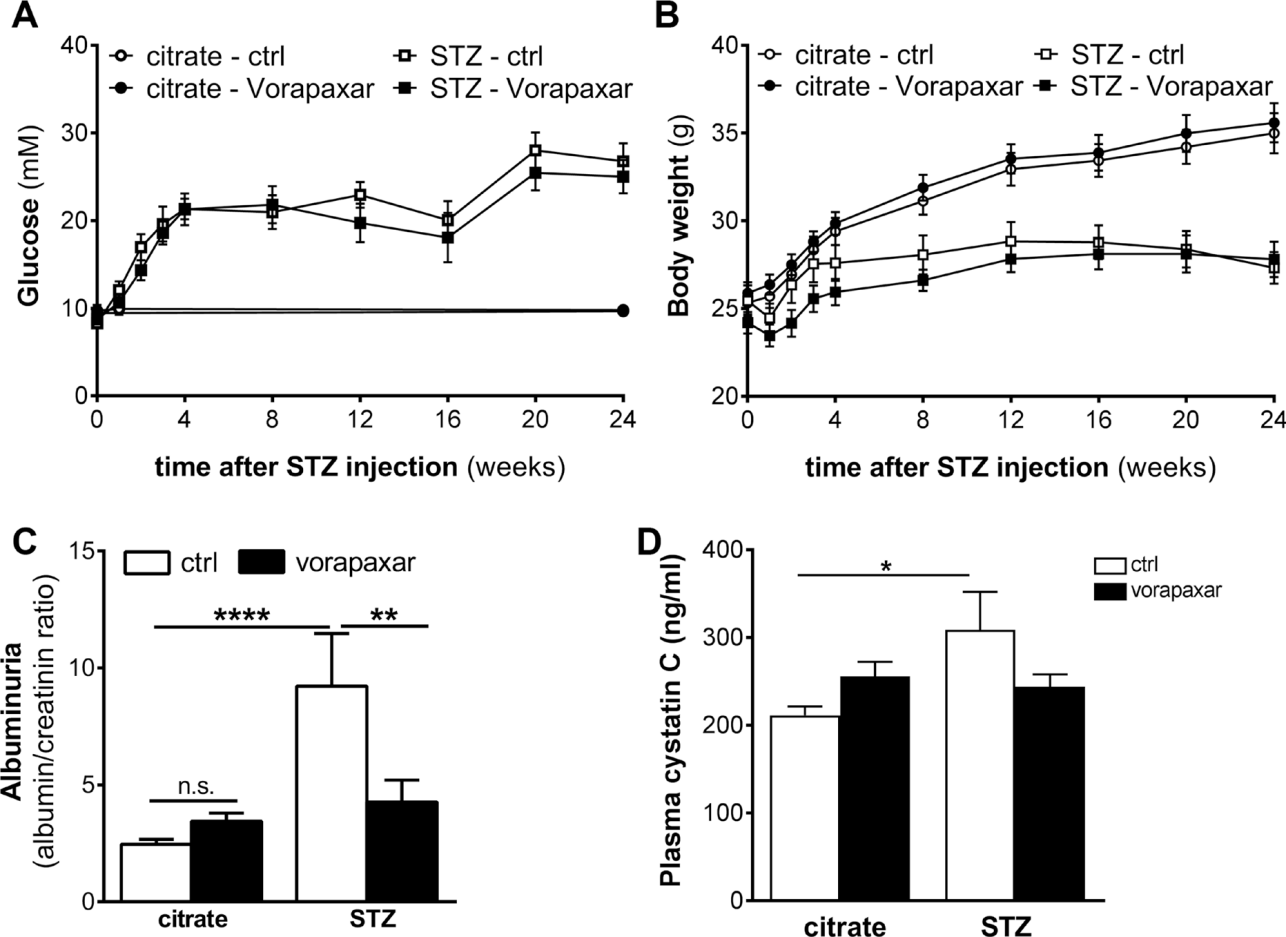
Reduced nephropathy after vorapaxar treatment in streptozotocin-induced diabetic mice (**A**) Glucose levels of control (citrate) and streptozotocin-induced (STZ) diabetic mice during control (ctrl) or vorapaxar treatment (**B**) Body weight of control (citrate) and streptozotocin-induced (STZ) diabetic mice during control (ctrl) or vorapaxar treatment (**C**) Albumin-to-creatinine ratio in urine and (**D**) plasma cystatin C levels in control and vorapaxar treated mice 24 weeks after streptozotocin (STZ) or citrate injections. Indicated is the mean ± SEM. Citrate ctrl, *n =* 11; citrate vorapaxar, *n* = 11; STZ ctrl, *n =* 6; STZ vorapaxar, *n =* 8. One-way ANOVA with Bonferroni post-hoc analysis and unpaired *t*-test were used, ^*^p < 0.05; ^**^p < 0.01; ^****^p < 0.0001.

Four weeks after the induction of diabetes by streptozotocin injections, both diabetic and non-diabetic mice were randomly assigned to vorapaxar or control treatment (*n =* 11 in all groups). Body weight and blood glucose levels were similar in vorapaxar and control treated mice during the course of the experiment (Figure 1A and 1B). As shown in Figure 1C, urinary albumin secretion was significantly increased after streptozotocin injections in control treated mice. Despite having similar glucose levels, no increase in albuminuria was observed in diabetic mice treated with vorapaxar. Similarly, the levels of plasma cystatin C were increased in control treated diabetic mice compared to nondiabetic mice. No increase upon diabetes was observed in the vorapaxar treated mice, showing that vorapaxar limits the diabetes-induced increase in plasma cystatin C (Figure 1D). Finally, no differences on inflammation were observed between control and vorapaxar treated mice (Supplementary Figure 1).

### Vorapaxar treatment reduces diabetes-induced mesangial expansion and glomerular fibronectin deposition

**Figure 2:**
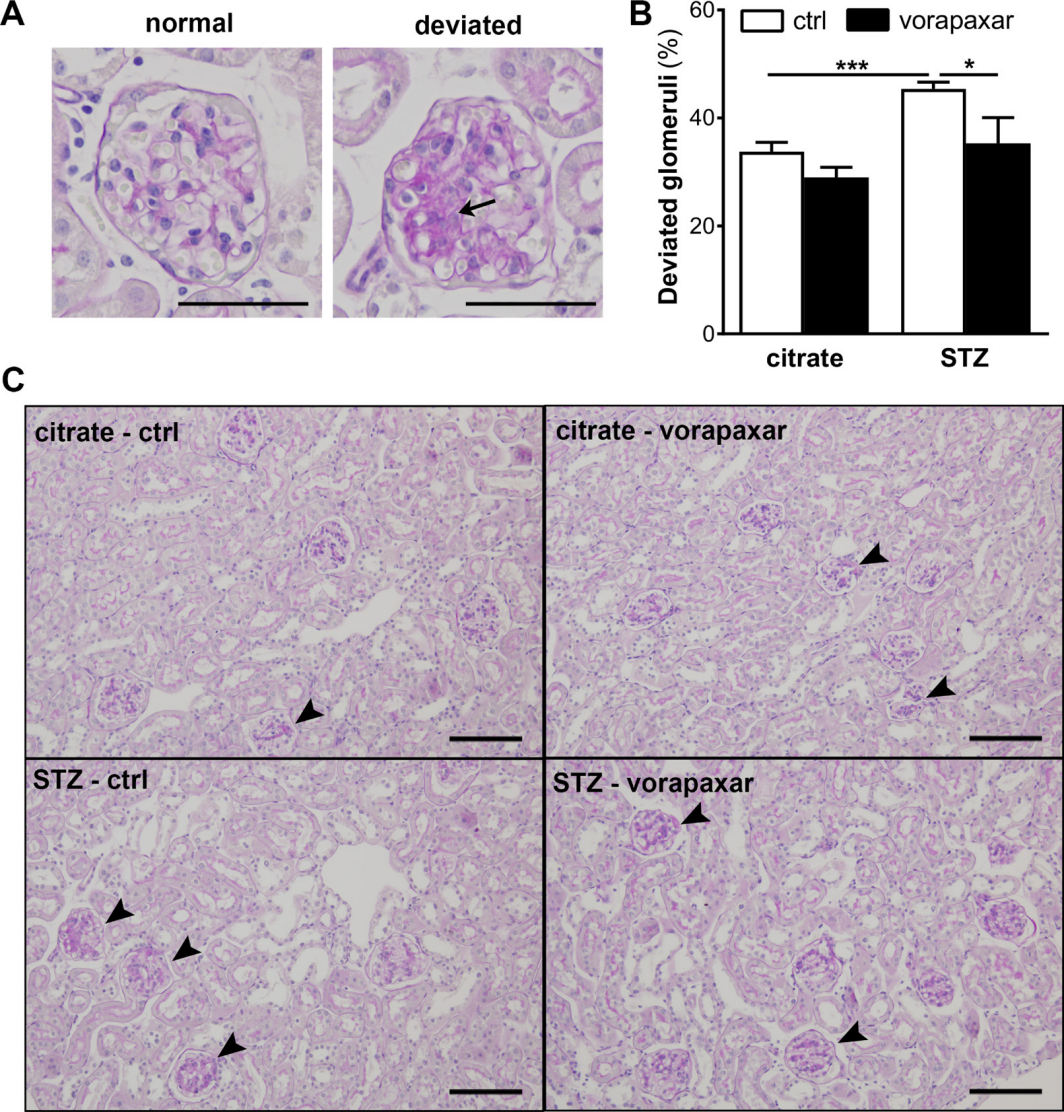
Limited mesangial expansion upon vorapaxar treatment in STZ-induced diabetic mice (**A**) Micrograph showing a PAS-D stained glomerulus scored ‘normal’ and ‘deviated’. The arrow pinpoints area of mesangial expansion. Scale bars: 50 µm (**B**) Quantification of mesangial expansion in glomeruli of diabetic (STZ) and non-diabetic (citrate) mice, upon control or vorapaxar treatment. Glomeruli were scored either normal or deviated as described in the materials and methods section. (**C**) Representative pictures of deviated glomeruli in control and vorapaxar treated diabetic mice. The arrowheads pinpoint glomeruli that were scored ‘deviated’. Scale bars: 100 µm. Citrate ctrl, *n =* 11; citrate vorapaxar, *n =* 10; STZ ctrl, *n =* 5; STZ vorapaxar, *n =* 8. One-way ANOVA with Bonferroni post-hoc analysis was used, ^*^p < 0.05; ^***^p < 0.005.

We previously showed that diminished albuminuria in PAR-1 deficient mice was accompanied by a reduction of mesangial expansion [19]. Consequently, we next evaluated the effect of vorapaxar on mesangial expansion in diabetic mice. An increase in mesangial expansion was observed in control treated diabetic mice as evident from an increase in the number of deviated glomeruli (Figure 2). Compared to control treatment, vorapaxar significantly reduced the number of glomeruli with mesangial expansion.

**Figure 3:**
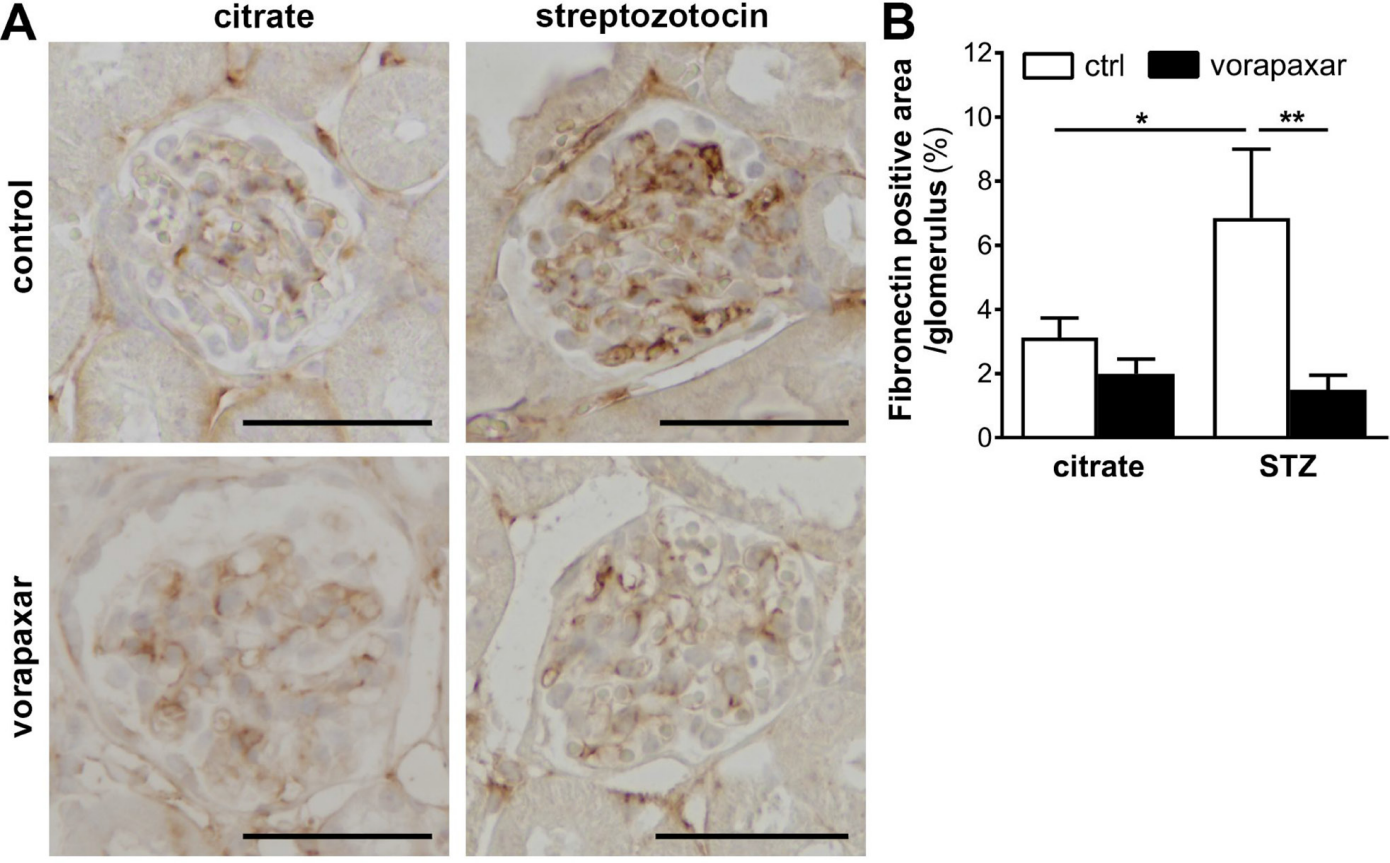
Limited glomerular fibronectin deposition upon vorapaxar treatment in STZ-induced diabetic mice (**A**) Representative pictures of fibronectin stained paraffin sections of kidneys of control and vorapaxar treated mice, 24 weeks after streptozotocin or citrate injections. Scale bars: 50 µm (**B**) Quantification of fibronectin deposition. Indicated is the mean ± SEM. Citrate ctrl, *n =* 10; citrate vorapaxar, *n =* 11; STZ ctrl, *n =* 5; STZ vorapaxar, *n =* 7. One-way ANOVA with Bonferroni post-hoc analysis was used, ^*^p < 0.05; ^**^p < 0.01.

Finally, glomerular fibronectin expression was assessed as PAR-1 directly induces fibronectin production by mesangial cells and fibronectin deposition was reduced in diabetic PAR-1 deficient mice as compared to control diabetic mice [19]. In line with our previous experiments, glomerular fibronectin expression was induced in diabetic control treated mice. Importantly however, no induction of fibronectin deposition was observed in vorapaxar treatment diabetic mice (Figure 3).

## DISCUSSION

Diabetic nephropathy is a major complication of diabetes which ultimately requires renal replacement therapy. Treatment options to delay or prevent the development of diabetic nephropathy are therefore eagerly awaited. We have recently shown that genetic ablation of PAR-1 limits the development of diabetic nephropathy in streptozotocin-induced diabetic mice by diminishing mesangial cell proliferation and extracellular matrix production. To evaluate the clinical potential of PAR-1 inhibition, we here treated diabetic mice with the PAR-1 antagonist vorapaxar. We show that inhibition of PAR-1 by vorapaxar treatment, started after the onset of diabetes, limits the development of diabetic nephropathy. In line with PAR-1 deficiency, vorapaxar treated mice developed less albuminuria, mesangial expansion and fibronectin deposition, as compared to control treated mice. Our data thus confirm the key role of PAR-1 in the pathogenesis of diabetic nephropathy and point to the potential clinical significance of vorapaxar treatment in diabetic patients.

Vorapaxar (SCH530349, Zontivity) is a PAR-1 antagonist, used for secondary prevention of atherothrombotic events in patients with previous myocardial infarction. In the TRA-CER trial [20] and the TRA 2° P-TIMI trial [21], treatment with vorapaxar led to reduced cardiovascular death, myocardial infarction, or stroke both in patients at high risk for cardiovascular events and in patients with a history of myocardial infarction, ischemic stroke, or peripheral arterial disease [22, 23]. Interestingly, treatment with vorapaxar appeared especially beneficial in patients with diabetes, with an absolute risk reduction for cardiovascular events of 3,5% (compared to 1,36% absolute risk reduction in non-diabetic patients) [24, 25].

Our data show that vorapaxar limits diabetic nephropathy in mice and suggest that vorapaxar may be pursued in patients. Importantly however, vorapaxar not only acts upon mesangial cells but (amongst others) also blocks PAR-1-dependent platelet aggregation which may lead to bleeding complications. Indeed, clinical trials using vorapaxar note an increased risk in bleeding and the TRA-CER trial was even terminated early due to excessive bleeding in patients on vorapaxar treatment (on top of aspirin and P2Y12 inhibitor) [22]. In a clinical setting, it should be critically evaluated whether kidney preservation by vorapaxar would outweigh the bleeding risk in individual patients. Ultimately, finding the PAR-1 agonist responsible for initiating the PAR-1 response in diabetic nephropathy, may lead to the development of a PAR-1 based treatment strategy avoiding the increased risk of bleeding.

In conclusion, vorapaxar treatment prevents the development of diabetic nephropathy in streptozotocin-induced diabetic mice, pinpointing PAR-1 as a novel therapeutic target to pursue in the setting of diabetic nephropathy.

## MATERIALS AND METHODS

### Mice

Wild type C57BL/6 mice were purchased from Charles River (Maastricht, the Netherlands). All experiments were approved by the Institutional Animal Care and Use Committee of the University of Amsterdam. All mice were maintained according to institutional guidelines. Animal procedures were carried out in compliance with the Institutional Standards for Humane Care and Use of Laboratory Animals of the Academic Medical Center. The Animal Care and Use Committee of the Academic Medical Center approved all experiments.

### Experimental diabetic nephropathy model

Eight to twelve week-old male wild type C57Bl/6 mice were injected intraperitoneally with streptozotocin (50 mg/kg body weight) or citrate for 5 consecutive days to induce diabetes. Four weeks after the induction of diabetes, mice were randomly assigned to control (*n =* 11) or vorapaxar (*n =* 11) treatment. Vorapaxar (1,75 mg/kg) was administered orally twice a week at 100 µl/10 g, whereas a similar amount of vehicle solution (0,02% hydroxyproline-β-cyclodextrin in 0,1% DMSO) was administered as control treatment. After 20 weeks of treatment, 24 h urine samples were collected using metabolic cages, after which the mice were sacrificed and blood and kidneys were harvested for further analysis. During the experiment, blood glucose levels were measured from tail vein blood using an Accu-Chek Aviva glucose meter (Roche). Five control treated mice and three vorapaxar treated mice did not develop diabetes (i.e. glucose levels remained below 15 mM) and were therefore excluded from further analysis. Plasma cystatin C (R&D systems) and urine albumin (Bethyl laboratories) levels were determined by ELISA according to the manufacturer’s instructions. Urine creatinine levels were determined using an enzymatic mouse creatinine assay kit (CrystalChem), according to the manufacturer’s instructions. Glomerular injury was determined by histological analysis and fibronectin deposition by immunohistochemical staining analysis.

### (Immuno)histopathology

Formalin-fixed, paraffin embedded, kidney slides were periodic acid–Schiff–diastase (PAS-D) stained following routine procedures. The extent of glomerular injury was determined by two independent observers in a blinded fashion. To quantify glomerular injury, 50 glomeruli per mouse, were scored as either normal or deviated. Glomeruli were scored as deviated when mesangial expansion was apparent as clusters of >3 mesangial cells.

Glomerular extracellular matrix accumulation was determined using goat-anti-fibronectin (1:500; sc-6953; Santa Cruz Biotechnology) antibody as described before [26]. In short, paraffin embedded slides were deparaffinized, and endogenous peroxidases were inhibited by 15 minutes incubation in 0,3% H_2_O_2_ at room temperature. Slides were boiled in citrate buffer (pH6.0) for 10 min, blocked with normal goat serum or Ultra V block (Thermo Scientific, Runcorn, UK) for 30 min, and incubated overnight with the primary antibody. Slides were incubated with HRP conjugated rabbit-anti-goat IgG (P0160; Dako) for 30 min at room temperature, visualized with DAB (BS04-999; Immunologic) and counterstained using haematoxylin. Slides incubated without the primary antibody were used as negative controls to exclude nonspecific binding of the secondary antibody. Pictures were taken at 20 times magnification using a Leica DM5000B microscope equipped with a Leica DFC500 camera and Image Pro Plus software (vs 5.02; Media Cybernatics). Fibronectin positive areas (expressed as a percentage) was determined, per glomerulus (expressing the density of ECM) using ImageJ software (U.S. National Institutes of Health, Bethesda, MD, USA) in 25 glomeruli per mouse.

### RNA isolation and RT-qPCR

For gene expression analysis, mRNA was isolated from kidney homogenates using TriReagent (#11667165001; Roche Diagnostics) according to the manufacturers recommendations. All mRNA samples were quantified by spectrophotometry and stored at −80° C until further analysis. One μg of mRNA was treated with RQ1 DNAse (M6101, Promega, Madison, WI, USA) and subsequently converted to cDNA using M-MLV reverse transcriptase (M1705, Promega, Madison, WI, USA) and random hexamer primers (#SO142, Fisher scientific, Landsmeer, the Netherlands) according to the manufacturers recommendations. qPCR and subsequent analysis were performed using sensiFAST No-ROX PCR master mix (GC Biotech) on a Lightcycler 480 machine and corresponding software (Software release 1.5.0 (1.5.0.39), Roche, Almere, the Netherlands). Expression levels were normalized using the average expression levels of HPRT1, GAPDH and TBP. The following primer sequences were used:

mIL6 forward: 5′- GCTACCAAACTGGATATAATCAGGA-3′ reverse: 5′- CCAGGTAGCTATGGTACTCCAGAA-3′; mTNFα forward: 5′- CTGTAGCCCACGTCGTAGC-3′ reverse: 5′- TTGAGATCCATGCCGTTG-3′; mMCP1 forward: 5′- CATCCACGTGTTGGCTCA-3′ reverse: 5′- GATCATCTTGCTGGTGAATGAGT-3′; mIL1β forward: 5′- TGAGCACCTTCTTTTCCTTCA-3′ reverse: 5′- TTGTCTAATGGGAACGTCACAC-3′; mTBP forward: 5′- CCTTGTACCCTTCACCAATGAC-3′ reverse: 5′- ACAGCCAAGATTCACGGTAGA-3′; mGAPDH forward: 5′- CTCATGACCACAGTCCATGC-3′ reverse: 5′- CACATTGGGGGTAGGAACAC-3′; mHPRT1 forward: 5′- CAGGCCAGACTTTGTTGGAT-3′ reverse: 5′- GACACAAACGTGATTCAAATCC-3′.

### Statistics

All values are expressed as mean ± SEM. All groups were tested for normality using the D’Agostino-Pearson omnibus normality test. Detected outliers were excluded from analysis. Differences between two groups were analysed using a *t*-test if data were normally distributed, or a Mann-Whitney *U*-test for non-parametric data. Multiple comparisons were analysed using one-way-ANOVA analysis or Kruksal-Wallis test (for nonparametric values), followed by Bonferroni’s or Dunns multiple comparison tests, respectively. All analyses were performed using GraphPad Prism version 5.01.

## SUPPLEMENTARY MATERIALS FIGURE



## Abbreviations

PAR: Protease-activated receptor
GPCR: G protein-coupled receptor
STZ: streptozotocin
TNF-α: Tumor necrosis factor-a
IL: Interleukin
HPRT1: Hypoxanthine-guanine phosphoribosyltransferase
GAPDH: Glyceraldehyde 3-phosphate dehydrogenase
TBP: TATA-binding protein
