# Supplementary material for: Vorapaxar treatment reduces mesangial expansion in streptozotocin-induced diabetic nephropathy in mice
Source: Oncotarget. 2018 Apr 24;9(31):21655–62. doi: 10.18632/oncotarget.25069 (PMC5955164; doi:10.18632/oncotarget.25069)
Supplement: Supplementary file 1 [file oncotarget-09-21655-s001.pdf]

Vorapaxar treatment reduces mesangial expansion in streptozotocin-induced diabetic nephropathy in mice

SUPPLEMENTARY MATERIALS

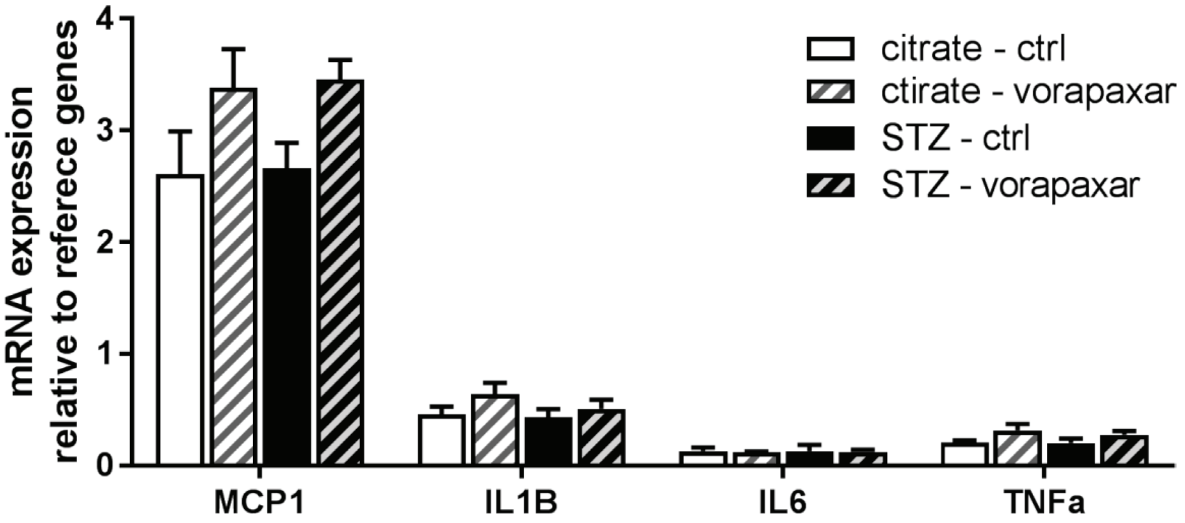

Supplementary Figure 1: Inflammatory mediators are unchanged upon vorapaxar treatment. Relative mRNA expression of MCP1, TNFα, IL1β and IL6 in non-diabetic control treated mice (citrate ctrl, white bars), non-diabetic vorapaxar treated mice (citrate vorapaxar, white/grey bars), diabetic control treated mice (STZ ctrl, black bars), and diabetic vorapaxar treated mice (STZ vorapaxar, black/grey bars). Indicated is the mean ± SEM.
